# Physical-Chemical and Microhardness Properties of Model Dental Composites Containing 1,2-Bismethacrylate-3-eugenyl Propane Monomer

**DOI:** 10.3390/biomimetics8070511

**Published:** 2023-10-27

**Authors:** Abdel-Basit Al-Odayni, Haifa Masfeer Al-Kahtani, Waseem Sharaf Saeed, Abdullah Al-Kahtani, Taieb Aouak, Rawaiz Khan, Merry Angelyn Tan De Vera, Ali Alrahlah

**Affiliations:** 1 Engineer Abdullah Bugshan Research Chair for Dental and Oral Rehabilitation, College of Dentistry, King Saud University, Riyadh 11545, Saudi Arabia; 2 Chemistry Department, College of Science, King Saud University, Riyadh 11451, Saudi Arabia; 3 Research Center, College of Dentistry, King Saud University, Riyadh 11451, Saudi Arabia; 4 Restorative Dental Sciences Department, College of Dentistry, King Saud University, Riyadh 11545, Saudi Arabia

**Keywords:** BisGMA, dental composites, depth of cure, eugenol, resin composite, Vickers microhardness, water sorption

## Abstract

A new eugenyl dimethacrylated monomer (symbolled BisMEP) has recently been synthesized. It showed promising viscosity and polymerizability as resin for dental composite. As a new monomer, BisMEP must be assessed further; thus, various physical, chemical, and mechanical properties have to be investigated. In this work, the aim was to investigate the potential use of BisMEP in place of the BisGMA matrix of resin-based composites (RBCs), totally or partially. Therefore, a list of model composites (CEa0, CEa25, CEa50, and CEa100) were prepared, which made up of 66 wt% synthesized silica fillers and 34 wt% organic matrices (BisGMA and TEGDMA; 1:1 wt/wt), while the novel BisMEP monomer has replaced the BisGMA content as 0.0, 25, 50, and 100 wt%, respectively. The RBCs were analyzed for their degree of conversion (DC)-based depth of cure at 1 and 2 mm thickness (DC1 and DC2), Vickers hardness (HV), water uptake (W_SP_), and water solubility (W_SL_) properties. Data were statistically analyzed using IBM SPSS v21, and the significance level was taken as *p* < 0.05. The results revealed no significant differences (*p* > 0.05) in the DC at 1 and 2 mm depth for the same composite. No significant differences in the DC between CEa0, CEa25, and CEa50; however, the difference becomes substantial (*p* < 0.05) with CEa100, suggesting possible incorporation of BisMEP at low dosage. Furthermore, DC1 for CEa0–CEa50 and DC2 for CEa0–CEa25 were found to be above the proposed minimum limit DC of 55%. Statistical analysis of the HV data showed no significant difference between CEa0, CEa25, and CEa50, while the difference became statistically significant after totally replacing BisGMA with BisMEP (CEa100). Notably, no significant differences in the W_SP_ of various composites were detected. Likewise, W_SL_ tests revealed no significant differences between such composites. These results suggest the possible usage of BisMEP in a mixture with BisGMA with no significant adverse effect on the DC, HV, W_SP,_ and degradation (W_SL_).

## 1. Introduction

Since the 1960s, resin-based composites (RBCs), which are composed of a resin matrix, fillers, and a matrix-filler coupling agent, have been the most widely utilized biomaterials to restore dental caries and other defects [1,2]. They are the first choice in restorative dentistry for patients and practitioners due to their aesthetics and fabrication simplicity. A resin matrix comprises crosslinking monomers, a photoinitiator system, and other additives forming a dense polymeric net upon photopolymerization [3]. Typically, crosslinkers (also called multifunctional monomers) play an essential role in the final properties of the composites and are primarily of (meth)acrylic type. Bisphenol A-glycidyl methacrylate (BisGMA) is the common base monomer in RBC’s matrix, favored due to its benefits to the resulting materials, including aesthetics, low shrinkage, and thermal stability [1,4]. However, its high viscosity brings some issues to the handling and application of the product. Furthermore, it prevents the use of high filler loads, which is necessary for restorative mechanical quality [5,6]. In addition to BisGMA, the matrix usually contains diluents to reduce the matrix’s viscosity and, thus, overcome the BisGMA-related drawbacks. The most commonly used diluent is triethylene glycol dimethacrylate (TEGDMA). However, TEGDMA has its own disadvantages, such as higher hydrophilicity and polymerization shrinkage, susceptibility to cyclization rather than crosslinking, and possible cytotoxicity [5,7]. As a result, researchers have investigated alternatives for BisGMA [8,9,10,11], either by modifying its structure or synthesizing new di(multi)functional analogs, targeting the matrix’s viscosity reduction to achieve the desired features of the final composite.

RBCs are the material of choice to restore minimal invasive cavities, not only because of their aesthetic qualities but also because of their biocompatibility and ability to adhere to tooth structures [12,13]. However, discoloration with time, poor marginal sealing, and degradation are the main disadvantages of their use and are directly related to their composition [14,15,16], including polymer matrix and filler content. Hence, the long-term existence of restorative materials in the oral environment necessitates a strong and stable product. The oral pH and temperature cycles may alter the composite components, resulting in filtration and reducing their durability [14]. Although the physical and chemical properties of the RBCs may be affected by solvent uptake, the two main concerns to be firmly taken into consideration when developing resin materials are the short-term release of uncured components and the long-term elution of degradation products [17,18]. The clinical performance of the dental material is highly influenced by water sorption; therefore, it plays a crucial role in deciding the clinical success, despite dental composite being considered stable and impermeable to water [19]. Water uptake is generally associated with the polymer network, which in turn is fostered by the chemical structure of the matrix [20,21]. Considering the structural properties of the composite components, the hydrophilic matrix has been reported to be the primary cause of water uptake [22]. The higher the hydrophilicity of the organic matrix, the greater the water uptake. The process may result in restorative discoloration, lower wear resistance, mechanical quality deterioration, the release of unreacted monomers, and hydrolytic degradation of bonds [13,19]. Conversely, solubility can contribute to discoloration and bulk weakening [23] of the dental restoratives. It is a sign of the low reactivity of the monomers used in the matrix, the degradation possibility brought on by the composite-making process, and the degree of hydrophobicity of the contents.

The conventional resin in the RBDs is commonly based on the monomers BisGMA, UDMA, TEGDMA, and others with mono-, di-, or multi-functionalities. However, BisGMA is the popular one, which presents at a higher rate than other monomers [24]. Chemically, BisGMA holds two hydroxyl groups, which supposedly drive its high viscosity and hydrophilicity [25]. As water diffuses into the restorative material, it may trigger chemical degradation, resulting in the formation of hydrolytic products. Hence, water also facilitates removing such degradation products and further contributes to water solubility via a releasing event. Water diffusion will also lead to the erosion of the organic matrix due to the release of unreacted monomers, which is more prominent in the early phase after restoration and will result in mass loss of the dental composite material [26].

The mechanical properties of dental materials determine how long they endure when used in the mouth [27]. Hence, flexural resistance and hardness are two of the most studied mechanical attributes because they closely resemble the forces generated during mastication and those supported by the material [28]. The hardness of RBCs is typically linked with the degree of conversion, which in turn depends on polymerization conditions and composite substances (type and quantity) [29,30]. It determines the material’s abrasion resistance, indirectly influencing bacterial adhesion by making surfaces more easily roughened [16,17]. The Vickers hardness test is a versatile method for measuring macro and microhardness, easy to carry out, and can be applied to small areas and various types of materials [31].

The 3-(4-allyl-2-methoxyphenoxy)propane-1,2-diyl bis(2-methylacrylate), shortened as 1,2-bismethacrylate-3-eugenyl propane (BisMEP), is a new synthesized dimethacrylated monomer containing eugenol moiety as a pendent group. The monomer was analyzed for its structural integrity and then incorporated in place of the BisGMA matrix of experimental RBCs. Then, the composites were characterized for their thermal stability, flowability, and degree of conversion [8], resulting in promising features to be investigated further. Hence, composite stability and mechanical withstanding are crucial properties of dental composites that could be targeted for analysis.

Eugenol is a versatile bioactive molecule and one aromatic building block for obtaining bio-based monomers [32]. Additionally, it has a bright history of use in medicine as an antimicrobial, antiseptic, and anesthetic agent [33], making it one essential precursor for several transformations, including the production of adhesives and polymerizable and non-polymerizable derivatives [32,34]. Therefore, many methacrylate-derivatives of eugenol have been synthesized and analyzed as adhesives, dental fillings, and orthopedic cements [34,35,36,37]. Indeed, eugenyl moiety is an attractive substance that could retain its bioactivity and function as an effective antimicrobial agent for particular applications, including cosmetics and dentistry [38]. Additionally, its allylic double bond enables further reactivity of functional polymers [32].

Recently, trials have been conducted to modify eugenol with polymerizable functional groups, predominantly of (meth)acrylate type, to be incorporated within resin matrices where moldable and sit-setting fabrication with long-live functioning is required [8,35,39]. In this project, BisMEP difunctional monomer was applied to incrementally substitute BisGMA in experimental RBCs to assess the effect of such replacement on the microhardness, depth of cure via degree of conversion, water sorption, and water solubility properties of photocured model composites. It is hypothesized that the replacement of BisGMA by BisMEP has no significant effect on (1) degree of conversion, (2) microhardness, (3) water sorption, and (4) water solubility. Furthermore, (5) there is no effect of curing thicknesses of 1 and 2 mm on the degree of conversion within the *p*-value of 0.05.

## 2. Materials and Methods

### 2.1. Chemistry-General

BisGMA (>98%), TEGDMA (>95%), camphorquinone (CQ, 97%), 2-(dimethylamino)ethyl methacrylate (DMAEMA; 98%), and (3-(trimethoxysilyl)propyl methacrylate (γ-MPS, 98%) were purchased from Sigma-Aldrich (Taufkirchen, Germany). Hexane (Hx, 95%) was bought from Avonchem, Macclesfield, UK. Ethyl acetate (EA, ≥99.5%) was purchased from Fisher Scientific, Loughborough, UK. BisMEP and silanized silica were synthesized as previously described [8,40].

### 2.2. Preparation of Model Composites

Four groups of experimental composites (CEa0, CEa25, CEa50, and CEa100) were formulated by mixing the organic components (BisGMA, TEGDMA, BisMEP, DMAEMA, and CQ) with the synthesized silanized silica fillers, as summarized in Table 1. The control group consists of BisGMA as the base resin, TEGDMA as the diluent monomer, and silanized silica as the filler. The test groups were prepared by replacing 25, 50, or 100 wt% BisGMA with BisMEP, the monomer of interest, to obtain CEa25, CEa50, and CEa100, respectively. Typically, the monomers were manually homogenized using a stainless-steel spatula. Then, the initiator system (CQ and DMAEMA as 0.2 and 0.8 wt% in reference to the total mass of the monomers) was dissolved in the monomer mixture. After the complete dissolution of CQ, the predetermined amount of the fillers was added in a portion with vigorous mixing. The composites were thus manually mixed using a spatula tool, then further homogenized using an asymmetric centrifugation technique in a TM DAC 150 FVZ speed mixer, Hauschild and Co. (Hamm, Germany), three times (for 1 min each and 2 min rest in between) at 3000 rpm. After that, the composites were vacuumed for 10 min at room temperature and then refrigerated at about 8 °C until used.

### 2.3. Degree of Conversion and Dept of Cure

The depth of cure of the photocured model composites (CEa0–CEa100) was assessed in terms of the degree of conversion using the FTIR technique; an attenuated total reflectance–Furrier transform infrared (ATR–FTIR) technique was employed with a Nicolet iS10 FTIR spectrometer from Thermo Scientific (Madison, WI, USA). For this, samples were packed in 5 mm diameter stainless-steel disks of 1 or 2 mm thickness, covered with plastic strips on either side of the mold followed by glass slides, and then light-cured from the top side for 1 min using an LED curing unit (Bluephase, Ivoclar Vivadent, Schaan, Liechtenstein) characterized with a light density of 650 mW/cm^2^, broad wavelength range of 385–515 nm, and an approximately 10 mm light guide tip. The FTIR spectra for the bottom side were collected before and after irradiation over the range from 650 to 4000 cm^−1^, with 16 runs per spectrum and a 4 cm^−1^ wavelength resolution. The DC was quantified by comparing the peak area of the polymerizable aliphatic C=C bonds (1638 cm^−1^) before and after curing in reference to the peak area of C-H bending at 1451 cm^−1^ in the matrix monomers [8,41], as given in Equation (1).

(1) $$\mathrm{DC}\left(\%\right)=\left[1-\frac{{\left(\frac{A_{1638}}{A_{1451}}\right)}_{\mathrm{cured}}}{{\left(\frac{A_{1638}}{A_{1451}}\right)}_{\mathrm{uncured}}}\right]\times 100$$

where *A*_1638_ and *A*_1451_ are the area at 1638 and 1451 cm^−1^, respectively.

### 2.4. Vickers Hardness Test

The hardness of the specimens was studied using a microhardness tester (INNOVATEST Europe BV, Maastricht, The Netherlands) equipped with a diamond indenter. Hence, disc-shaped 5 mm diameter and 2 mm thickness samples were prepared (photo-cured from one side for 60 s, as above), and after 10 min, samples were moved to plastic containers and conditioned at 37 °C humified environment for 24 h before testing. The measurement was carried out using 200 gf as a loading force and 15 s as a dwell time for three replicates, with three readings per specimen selected at a distance of at least 1 mm from each other. The model composites’ mean Vickers hardness number (VHN) values were calculated using the machine software based on the formula in Equation (2). The indentation was monitored with the 40× magnification lens of the microscope.

(2) $$\mathrm{VHN}=1.854\left(\frac{F}{D^{2}}\right)$$

where *F* and *D*^2^ are the applied load (kilograms-force) and the indent area (mm^2^), respectively.

### 2.5. Water Uptake and Solubility

Water sorption and water solubility of the examined model composites were assessed in distilled water [37,42] to simulate the oral environment. Hence, disc-shaped specimens (15 mm diameter, 2 mm thickness, n = 3) were fabricated in stainless-steel molds and light-cured using a 10 mm diameter-tip curing unit as above. After disc preparation, a drying-swelling-drying process was performed. Typically, the discs were dried in a desiccator containing anhydrous potassium sulfate maintained at 37 ± 2 °C. After every 24 h, the desiccator containing the test discs was transferred to cool at room temperature for about 2 h, then the specimen dry weight was recorded. When the dry weight was unchanged (termed *m*1), samples were immersed in the distilled water and incubated in the oven at 37 ± 2 °C for the water sorption test. Every 24 h, the disk temperature was brought to room temperature. Then, the samples were carefully taken from the swelling water, gently swabbed, weighed again, and returned to the water, and the attained constant weight (*m*2) was considered the maximum swell. To assess the solubility of the materials, the swollen discs were dried again until constant weight (*m*3) was attained, as conducted with m1. W_SP_ and W_SL_ from three replicates were calculated using Equations (3) and (4).

(3) $$W_{\mathrm{SP}}(\%)=\left(\frac{m2-m1}{m1}\right)\times 100$$

(4) $$W_{\mathrm{SL}}(\%)=\left(\frac{m1-m3}{m1}\right)\times 100$$

### 2.6. Statistical Analysis

Statistical analysis was conducted using IBM SPSS statistics version 21 (IBM Corp., Armonk, NY, USA). One-way analysis of variance (ANOVA) followed by Tukey post-hoc test and paired samples *t*-Tests were used for evaluation, and a *p*-value of less than 0.05 was considered significant. Figures were prepared using Origin 2018 software (OriginLab Corporation, Northampton, MA, USA) for the mean ± standard deviation of five replicates.

## 3. Results and Discussion

### 3.1. Characterization

**Figure 1:**
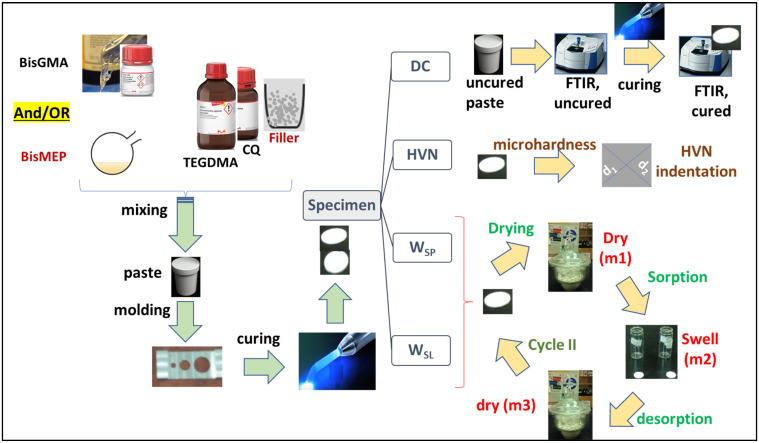
Schematic illustration for resin-based composite preparation and curing degree (DC), microhardness (HV), water sorption (W_SP_), and water solubility (W_SL_) assessment routes.

**Figure 2:**
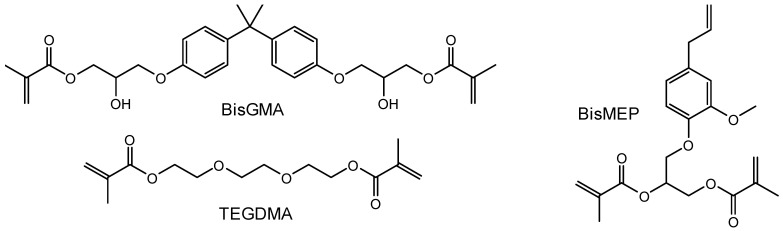
Chemical structure of BisGMA, TEGDAMA, and BisMEP monomers.

Figure 1 illustrates the working experimental route, including synthesis, characterization, and applications. The structural integrity of the BisMEP and synthesized silanized silica (S-SiO_2_) was confirmed and reported previously [8,40]. The chemical structures of matrix monomeric components are shown in Figure 2. BisGMA was used as the main resin, TEGDMA was the diluent, and BisMEP was the newly introduced monomer to be tested, which, as can be seen, is incrementally employed in place of BisGMA in the target composites CEa0–CEa100. By comparing the structural properties of BisGMA and BisMEP, one can see that BisGMA is unbranched, has an aromatic core structure of bisphenol A, and involves two hydroxyl groups. This semi-linearity and the presence of two OH groups drive its high viscosity by providing strong intermolecular H-bonding interaction between molecules. BisMEP, on the other hand, has a lower molecular weight (374.4 g/mol) than BisGMA (512.6 g/mol) and has no ability to create H-bonding. Under similar conditions, BisMEP viscosity (0.379 Pa·s) is remarkably lower than that of BisGMA (580.977 Pa·s). According to the previous study [8], composites incorporating BisMEP, which partially replaced BisGMA (up to 50 wt%) in matrices containing 50 wt% TEGDMA, exhibited better rheological properties and comparable DC to composites with BisGMA-unreplaced matrix. The effect of such substitution of BisGMA by BisMEP on the composite’s DC, depth of cure, microhardness, water sorption, and water solubility were intended to be investigated in this work.

### 3.2. Analysis of Curing Degree

The DC at the bottom side of the photocured CEa composites with 1- and 2-mm disc thicknesses are given in Table 2, symbolled as DC1 and DC2, respectively. As seen among the tested composites, the DC decreases as BisMEP content increases. Thus, DC1 insignificantly differs due to the replacement of BisGMA by BisMEP up to 50% (CEa50). However, the difference became significant as BisMEP completely replaced BisGMA (CEa100), so the first hypothesis was rejected. This result agrees with the previous report, in which the DC was analyzed for the cured side of the disk with 1 mm thickness [8]. Analysis of DC2 indicated an earlier inhibitory effect of the curing process than DC1 due to incorporating a high quantity of BisMEP, which becomes significant above CEa25, thus confirming the rejection of the first hypothesis in such a case. By comparing the DC at 1 and 2 mm thickness, DC1 and DC2, respectively, it is found that there are no significant differences between them at low dosages of BisMEP (i.e., CEa0 and CEa25). Indeed, BisMEP is a low viscosity monomer (0.379 Pa·s) compared to BisGMA (580.977 Pa·s), a character that supports increasing DC; on the other hand, the structural properties of BisMEP may retain a bit inhibitory characters of eugenol-moiety, causing reduction of DC [8]. Hence, it seems that the two influencers have contributed to balancing of DC close to each other at a low BisMEP quantity. However, in the incorporation of high quantity, e.g., close to that in CEa50 and CEa100, the inhibitory effect dominates, resulting in a significant drop in the values of DC. According to the literature, there is no consensus regarding the minimum DC required for most restoratives, but a minimum value of 55% was suggested as suitable for clinical approaches [43]. In the current course, the DC was found to be higher than 55%, the minimum limit, for CEa0–CEa50 at 1 mm thickness (DC1) and CEa0–CEa25 at 2 mm thickness (DC2), which supports the possible incorporation of BisMEP in place of BisGMA up to 25%. The 2 mm increment thickness is reported as the gold standard for placement and curing of composite [44], despite manufacturers competing on introducing materials with a higher depth of cure to address the issues correlated with the 2 mm curing process, including time-consuming and technique sensitivity.

### 3.3. Vickers Hardness

The result of Vickers microhardness for the investigated model composites (CEa0–CEa100) is summarized in Table 2. As can be seen, the microhardness is insignificantly decreased with BisMEP increases (*p* > 0.05), from 50.04 ± 1.23 for the control (CEa0) to 46.66 ± 3.74 for CEa50. However, the difference became statistically significant (*p* < 0.05) for the composite with the complete replacement of BisGMA by BisMEP (CEa100); therefore, the second hypothesis was partially rejected. This result is mainly associated with composite materials and their DC. The DC was slightly but insignificantly reduced as BisMEP replaced BisGMA up to 50%; by approaching CEa100, the DC differs significantly from the control (CEa0). Hence, the microhardness varies with the DC; the viscosity differences between BisGMA and BisMEP could be the leading influencers. The viscosity of BisMEP was found to be more than 1500 times less than that of BisGMA [8]; the viscosity of CEa100 is more than 16 times lower than that of CEa0. The viscosity is structure-dependent; thus, as the hydrogen bonding associated with BisGMA is absent in BisMEP (Figure 2), the viscosity of the latter is less. A closer look reveals that the historical inhibitory effects of eugenol against free radical polymerization may still be a bit retained, which could explain why the DC drives the process rather than viscosity, as was previously claimed [8].

### 3.4. Water Sorption and Solubility

The data obtained for W_SP_ and W_SL_ of the investigated model composites are listed in Table 2 as well. As can be seen, there is no significant effect (*p* > 0.05) of replacing BisMEP in place of BisGMA on W_SP_ and W_SL_, thus accepting the third hypothesis. W_SP_ was insignificantly reduced from 2.39 ± 0.25 to 2.03 ± 0.77 wt% as replacement approached from CEa0 to CEa100. W_SL_ was found to be increased but within the proposed statistically insignificant range (*p* < 0.05) and supported accepting the fourth hypothesis. These findings suggested material-dependent behavior by showing the tendency of the composites to uptake less water and leach more substances as BisMEP quantity increases in the composite. The decrease in W_SP_ is supposedly a result of hydrophilicity reduction due to the replacement of BisGMA (high hydrophilic monomer) by BisMEP (low hydrophilic monomer). On the other hand, as there are no significant differences between test composites (CEa25–CEa100) and control (CEa0) even after the total replacement of BisGMA (CEa100), other factors may also participate in the W_SP_ mechanism. It could be suggested that the effect of TEGDMA is dominant, thus balancing the hydrophilicity decrease due to BisGMA replacement by the less hydrophilic monomer BisMEP.

W_SL_ is mainly associated with DC. Accordingly, the DC was insignificantly decreased as BisMEP developed from CEa0 to CEa50; however, by complete replacement of BisGMA by BisMEP in CEa100, the DC differed significantly from that of the control (BisMEP-free composite, CEa0). The DC is somehow affected by eugenol moiety, both their free radical scavenging and viscosity. Therefore, the hydrophobicity of BisMEP may support less water uptake, while its lower DC promotes water diffusion. The latter case may be the cause of an insignificant decreasing trend in W_SP_.

The process and impact of water sorption on RBCs can be illustrated based on the physical and chemical properties of both organic and inorganic components. Basically, hydrophilic organic molecules ‘resins’ have a high affinity to water. Thus, sorption and solubility occur when they come in contact with saliva ‘water’. During this process, water diffuses into the material, causing gradual expansion ‘swelling’. Then, swelling promotes hydrolysis ‘degradation’ as well as diffusion ‘leaching’ of hydrolysate and unreacted monomers, respectively.

The overall results for DC, depth of cure, W_SP_, and W_SL_ could be discussed in terms of the chemical structure of BisGMA and BisMEP. Hence, BisGMA molecular weight is higher and is more hydrophilic than BisMEP, a property that drives its high viscosity compared to BisMEP. On the other hand, BisMEP holds an eugenyl pendent group, which may decrease the molecular freedom during polymerization reactions. Even though the viscosity of BisMEP is low and could support higher DC, the inhibitory effect of eugenyl moiety via radical scavenging may be retained. Therefore, the insignificant difference in the DC due to the addition of BisMEP may be balanced by these two opposite characters, i.e., reduced viscosity and free radical inhibitory effect. Indeed, the DC affects most other properties, including material hardiness, water uptake, and water solubility. Therefore, close trends to that of DC among the investigated composites were observed. i.e., with VHN, W_SP_, and W_SL_, as discussed above. The results support no change in the composite properties when BisGMA is replaced by BisMEP up to ca. 25 wt%, featuring possible improvement of dental composite eugenol moiety.

## 4. Conclusions

Four model composites consisting of 66% silanized silica as fillers and 34% BisGMA/TEGDMA (1:1) as matrices were prepared, while BisGMA was replaced by 0.0, 25, 50, and 100% BisMEP. Based on the data obtained, the investigated BisMEP monomer can be added to a dental composite approximately up to 25 wt% of the total matrix contents to benefit the composite with handling properties without compromising its primary desired properties, including DC, mechanical hardness, W_SP_, and W_SL_. The DC-based depth of cure was statistically the same at 1 and 2 mm thickness. The swelling and degradative properties of the investigated composites in distilled water have proven minimal and statistically insignificant differences compared to the control (BisMEP-free composite, CEa0). Such findings support the possible incorporation of BisMEP monomer as a diluent for BisGMA in the resin-based dental composite. However, eugenol moiety is attractive to be further analyzed as a potential contact-active antimicrobial, making a case that is open for subsequent study.

## Figures and Tables

**Table 1:** Composition of the prepared resin-based model composites (CEa0–CEa100).

Composite (Group)	Filler, 66 wt%	Matrix, 34 wt%		
	Silanized Silica	TEGDMA	BisGMA	BisMEP
CEa0	66.00	17.00	17.00	0.00
CEa25	66.00	17.00	12.75	4.25
CEa50	66.00	17.00	8.50	8.50
CEa100	66.00	17.00	0.00	17.00

CQ and DMAEMA were added to the matrix as 0.2 and 0.8 wt% per total resin mass.

**Table 2:** Vickers microhardness (VHN), water sorption (W_SP_) and water solubility (W_SL_), and degree of conversion at 1- and 2-mm depth of the model composites CEa0, CEa25, CEa50, and CEa100.

Composite	VHN: Mean (SD); n = 5	W_SP_ (%): Mean (SD); n = 3	W_SL_ (%): Mean (SD); n = 3	DC1 (%): Mean (SD); n = 5	DC2 (%): Mean (SD); n = 5
CEa0	50.04 ^a^ (1.23)	2.39 ^a^ (0.25)	0.97 ^a^ (0.35)	60.28 ^a,A^ (5.54)	59.19 ^a,A^ (3.71)
CEa25	48.33 ^a^ (2.23)	2.19 ^a^ (0.21)	1.07 ^a^ (0.44)	58.72 ^a,c,A^ (7.40)	58.48 ^a,A^ (1.77)
CEa50	46.66 ^a^ (3.74)	2.13 ^a^ (0.91)	1.20 ^a^ (0.64)	57.04 ^a,c,A^ (5.65)	44.83 ^b,B^ (7.17)
CEa100	43.12 ^b^ (1.55)	2.03 ^a^ (0.77)	1.77 ^a^ (0.24)	49.50 ^b,c,A^ (4.16)	42.93 ^b,A^ (10.14)

Within the column, the different lowercase letters mean significant differences, *p* < 0.05. Within the row, the different uppercase letters mean significant differences between DC1 and DC2 of the same composite.

## Data Availability

The data presented in this study are available within the article.
